# Effects of a culturally specific tobacco cessation intervention among African American Quitline enrollees: a randomized controlled trial

**DOI:** 10.1186/s12889-017-5015-z

**Published:** 2018-01-10

**Authors:** Monica Webb Hooper, Kelly Carpenter, Michael Payne, Ken Resnicow

**Affiliations:** 1 Case Western Reserve University School of Medicine, Case Comprehensive Cancer Center, 11000 Euclid Ave, Cleveland, OH 44106-7285 USA; 2 Center for Wellbeing Research, Alere Wellbeing, 999 Third Avenue, Suite, Seattle, WA 1800 USA; 3 University of Michigan, 1415 Washington Heights, Ann Arbor, MI USA

**Keywords:** Smoking, Tobacco, Cessation, Culturally specific interventions, African Americans, Video-based interventions, Disparities

## Abstract

**Background:**

African Americans suffer disproportionately from tobacco-related illness and have more difficulty quitting smoking than other racial/ethnic groups. Previous research indicates that African American treatment-seekers are high utilizers of tobacco quitlines, yet cessation rates via quitlines are lower relative to whites. The goal of the present study is to test the effectiveness of adding a culturally specific, video-based, adjunct to standard quitline care. It is hypothesized that the integration of an evidence-based intervention (*Pathways to Freedom: Leading the Way to a Smoke-Free Community©; PTF*) into quitline services will increase cessation and treatment engagement compared to control conditions, and that effects will be moderated by sociocultural factors (e.g., culturally specific intervention expectancies, acculturation, and ethnic identity).

**Methods:**

This study is a 3-arm semi-pragmatic randomized controlled trial (RCT). Participants will be 1050 enrollees in the North Carolina State quitline (QuitlineNC) who self-identify as African American. Usual quitline care includes up to 4 proactive quit coaching calls, website access, and two-weeks of nicotine patch therapy. Eligible study participants will be randomized to receive (1) standard quitline services plus *PTF (PTF)*; (2) quitline services plus a standard tobacco cessation DVD (attention control); or (3) quitline services alone (usual care). Assessments will be conducted at baseline, 3 and 6-months post-enrollment. The primary outcome will be biochemically verified 7 day ppa at 6-months. Generalized linear mixed models (GLMMs) and hierarchical logistic regression will be used to assess the effects of treatment group on cessation outcomes and to test potential moderators.

**Discussion:**

This study will answer questions regarding the implementation and effectiveness of integrating a culturally specific video intervention into a real-world, population-level tobacco intervention. It will also aid our understanding of individual-difference variables that are associated with success. If an incremental benefit is found, this trial will have implications for increasing the responsiveness of tobacco quitlines for African Americans, reducing tobacco cessation disparities, and best practices for improving minority health. In addition, the *PTF* intervention has the potential for widespread disseminated through quitlines, which are available across the United States.

**Trial registration:**

Clinicaltrials.gov NCT03064971. Registered on February 22, 2017.

## Background

Racial disparities in tobacco control persist. African American adults are significantly more likely to report current smoking and have higher death rates from related illnesses relative to Whites [1]. This population is also less likely to achieve long-term tobacco use abstinence [2–4]. Multiple evidence-based interventions for cessation exist (e.g., face-to-face counseling), however their ability to provide cost-effective help on a mass scale is limited. Tobacco quitlines represent a population-based method of treatment delivery and serve more smokers per year than any other single-channel for moderate to intense counseling. Quitline counseling alone doubles the likelihood of cessation, and triples when combined with medication [5]. Quitlines are also important because they reduce barriers to care such as cost, health insurance, lack of transportation and/or childcare, and serve traditionally underserved populations, including ethnic/racial minorities and those living in rural settings [6].

Evidence suggests that African American smokers are interested in quitline services. Zhu et al. [7] found that compared to Whites, African American smokers are more likely to seek quitline services, and request counseling. Recent findings from the National American Quitline Consortium (NAQC) indicate that 18.7% of US quitlines callers self-identify as Black/African American which equals about 58,500 tobacco smokers each year [8], while being 13% of the U.S. population and 19% of smokers [1]. Few studies have reported outcomes by race/ethnicity, however, the extant literature demonstrates a lower likelihood of cessation among African American versus white quitline enrollees [9, 10]. It may be possible to enhance the efficacy of quitline services via culturally specific interventions, which integrate distinct social factors, such as history, beliefs, norms, smoking patterns, and cultural values and assets into the content. Culturally specific interventions are framed within a cultural context and are based on theory, empirical evidence, and characteristics of the target population [11, 12].

The literature suggests that culturally specific interventions are beneficial among some African American tobacco smokers. Research has found a general preference for culturally specific compared to standard interventions [13], and greater efficacy when delivered in a group intervention format [14]. However, given the heterogeneity within racial categories, the efficacy of culturally specific interventions may vary by individual differences [15]. The only published quitline study focused on African Americans found that culturally tailored counseling plus culturally specific written materials led to greater intervention satisfaction and quit attempts, yet effects on abstinence were less clear [16]. Given the disparity in cessation between African Americans and other racial groups, interventions with high scalability, and that reduce barriers to participation (e.g., accessibility, cost, health literacy, relevance), are needed. To this end, we developed and tested a video-based, culturally specific intervention designed for African American tobacco smokers, titled *“Pathways to Freedom: Leading the Way to a Smoke-Free Community”©(PTF)* [17].

The video-based *PTF* retains the framework of an earlier print-based *PTF* guide, which was guided by a model of community competence [18]. The video version is a substantial enhancement of the original guide, and translates the content of an efficacious cognitive behavioral therapy for smoking cessation adapted for African Americans [14]. The *PTF* video expands on topics such as the history of tobacco and African Americans, targeted tobacco industry marketing, mentholated cigarettes, the use of religion/spirituality as a coping strategy, and pharmacologic cessation aids. It utilizes cognitive behavioral strategies including cognitive restructuring and coping skill development, and highlights cultural assets. A 2-arm randomized trial demonstrated positive effects of *PTF* delivered as a digital video disc (DVD) compared to a standard control DVD [17]. Specifically, *PTF* was preferred and increased readiness to quit and risk perceptions.

### The present study

Despite the disparate tobacco use and illness rates, little attention has been placed on testing new behavioral approaches aimed at increasing quit rates among African American tobacco smokers. This randomized controlled trial (RCT) will seek to obtain real-world evidence for the efficacy of the *PTF* intervention as an enhancement to standard quitline counseling. The three aims of the study are to: (1) examine the effects of implementing a culturally specific, video-based intervention targeting African Americans in a state tobacco quitline; (2) examine the effect of *PTF* on treatment engagement; and (3) assess the feasibility of delivering *PTF* in an online format (exploratory). We hypothesize a positive effect of cultural specificity, such that the *PTF* intervention will demonstrate superior outcomes compared to the control conditions. The primary abstinence outcome will be cotinine-verified 7-day point-prevalence abstinence (ppa) at the 6-month follow-up. Secondary outcomes will include 7-day ppa at 3-months and treatment engagement. We will also examine ethno-cultural individual differences as moderating variables, including expectancies for culturally specific interventions, acculturation, and experiences of racial/ethnic discrimination.

## Methods/design

**Fig. 1:**
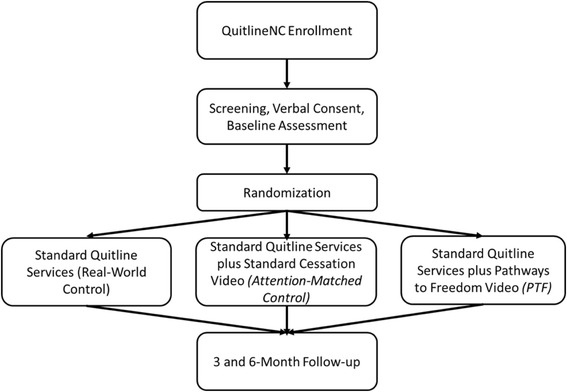
Study Design

This study is a 3-arm semi-pragmatic randomized controlled trial (RCT) [19] that includes elements of both implementation and effectiveness research [20]. Pragmatically, recruitment, enrollment, and intervention delivery are occurring in a real-world setting (the quitline), and inclusion/exclusion criteria are minimal to increase access to care and study generalizability. Participants are randomly assigned to receive one of 3 interventions (Fig. 1): standard care plus *PTF* video, standard care plus standard smoking cessation video, or standard care only—all supplemented by 2-weeks of NRT (the standard supply offered through the quitline). To increase accessibility, participants in the video conditions are mailed a DVD and have access to a private YouTube channel for online viewing of their respective video. Assessments will occur at baseline, 3, and 6-months post-enrollment. Thus, the study will answer whether standard quitline counseling can be enhanced by the provision of a culturally specific video-based intervention.

### Participants

Participants are African American tobacco smokers recruited from the North Carolina State Quitline (QuitlineNC; *N* = 1050). Inclusion criteria include (1) self-identification as Black/African American; (2) current smokers (smoked at least 100 lifetime cigarettes and currently smoke on some or every day) who would like help quitting; (3) eligible for state quitline services; (4) over 18 years of age; (5) access to devices that play DVDs or the internet; and (6) permanent contact information. Exclusion criteria are: (1) recent quitters (have not smoked in the past 7 days), (2) planning to move within the next 6 months, and (3) not ready to set a quit date within 30 days.

### Randomization

The individual is the unit of randomization. The randomization scheme was developed using a computer-generated random numbers table. We are using stratified random sampling (by sex) with equal allocation to conditions. Randomization occurs through an automated algorithm after Quit Coaches® indicate within the computer system that a participant has provided verbal consent. Participants are blind to their intervention condition.

### Procedures

During initial quitline enrollment, callers who self-identify as Black/African American are screened for eligibility. Eligible enrollees are offered the opportunity to participate in the study, and those who agree are transferred to a research-trained quitline coach to provide verbal informed consent and complete the baseline measures. Participants are then randomized to a study condition, and then begin standard care services from the quitline (described below). Participants in the *PTF* or attention-control conditions will receive the assigned video in DVD format by mail, in addition to a text, email and/or mailed letter including the link to a private YouTube channel for online viewing. Monthly promotional items, and bi-weekly reminders serve as prompts to encourage use of the videos and/or utilize the quitline services. Incentives for assessment completion include $15 at baseline, $25 at the 3-month follow-up, $60 for the 6-month follow-up, and $20 for returned saliva samples. Participants can self-withdraw from the study by informing the study by telephone or in-writing.

#### Interventions

**Fig. 2:**
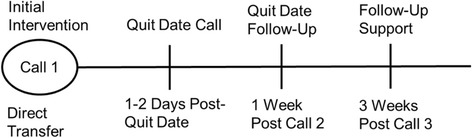
Quit Coaching Call Schedule

QuitlineNC is operated by Alere Wellbeing (owned by Optum), the leading provider of tobacco quitline services in the U.S. who delivers more than 350,000 coaching calls per year via 25 state quitlines and numerous commercial contracts. Beyond the initial counseling session, which occurs at baseline, participants may receive up to three additional proactive (i.e., Quit Coach initiated) counseling calls (Fig. 2), each designed to provide practical expert support to help participants develop problem-solving and coping skills, secure social support, and design a plan for successful cessation and long-term abstinence. Participants can also contact the quitline as needed for support between proactive calls. A 2-week “starter” pack of nicotine patches, which is consistent with standard quitline care, is provided at no-cost to participants per manufacturer dosing and usage instructions.

##### Standard Quitline services (real-world control)

During the initial counseling session, Quit Coaches® work with participants to set a quit date and develop a personalized plan for quitting. Subsequent coaching calls focus on cessation-related challenges and/or the use of NRT. Quit Coaches® review and modify individualized quit plans, and provide support as appropriate. If participants have an unsuccessful quit attempt, Quit Coaches assist with planning a new quit date and/or resolving ambivalence. For individuals who have successfully quit, coaches focus on relapse prevention. Participants also receive standard self-help materials by mail, which is often referred to by Quit Coaches.

##### Standard Quitline services plus standard cessation video (attention-matched control)

This condition includes standard care provided by QuitlineNC, plus a standard smoking cessation video (DVD and online). The evidence-based “*How to Quit”* DVD [21] contains 60 min of smoking cessation information and strategies. It describes the process of quitting, and strategies for increasing physical activity and healthy nutrition. It includes testimonials from former smokers and dialogue among smokers in a group counseling setting. The information is presented in a standard format, intended for the general population of smokers. Quitline counselors are trained to refer participants to appropriate sections for additional help. This condition is included to control for attention effects.

##### Standard Quitline services plus PTF (PTF)

The experimental intervention will include the standard care provided by QuitlineNC, with the addition of *PTF* video (DVD or online). The content of the intervention parallels the types of education, advice, and cessation/relapse prevention strategies that are delivered in clinic and quitline contexts, except for the focus on the specific needs of the African American community. The 60-min video describes the history of tobacco and African Americans, the health consequences of tobacco use and second hand smoke, strategies for cessation and relapse prevention, and mobilizing the community to fight against the tobacco industry. The culturally specific elements (e.g., focus on targeted tobacco industry marketing, menthol tobacco products, concerns about pharmacotherapy, religion/spirituality, family and community, African American images and music, etc.) are infused throughout the video. Quit Coaches® are trained to refer participants to appropriate sections for additional help.

##### Online access

Participants in both video conditions will have the opportunity to view their assigned video via the Internet. The goal is to increase accessibility of the intervention and allow viewing on mobile devices. We will text, email (and mail) a link to participants allowing them to access their assigned video using a private YouTube channel. Participants will be able to select sections for viewing.

### Measures

#### Baseline (Table 1)

Standard quitline enrollment items include demographics, tobacco use history (age of initiation, current smoking, daily smoking, types of products used, history of quit attempts, prior use of quitline/telephone counseling services), time to first cigarette, health insurance, chronic medical conditions (e.g., asthma, cancer), stage of readiness to quit, and electronic cigarette use. Study-specific measures include readiness to quit [22], perceived stress [23], depressive symptoms [24], acculturation [25], ethnic identity [26], ethnic discrimination scale [27], culturally specific intervention expectancy, and social support [28]. Measures are minimized to reduce participant burden.

**Table 1:** Study Specific Data Collection

Measure	Baseline	3-Month Follow-Up	6-Month Follow-Up
Demographics	X		
Tobacco Use	X		
Weight concerns	X		
Readiness to Quit	X	X	X
Perceived Stress	X	X	X
Depressive Symptoms	X	X	X
Acculturation	X		
Ethnic Identity	X		
Expectancies for Culturally Specific Interventions	X		
Perceived Social Support	X	X	X
Perceived Ethnic Discrimination	X		
Treatment Engagement		X	X
Treatment Utilization		X	X
TNP Use		X	X
Intervention Ratings		X	
Time-Line Follow-Back (smoking pattern)		X	X
Cotinine^a^			X

^a^Saliva for cotinine analysis is collected from self-reported abstainers only

#### Follow-up (3, and 6-months, Table 1)

Assessments are completed via telephone approximately 3 and 6-months after study enrollment (Fig. 1). Measures include treatment engagement [29], number of coaching calls completed, intervention ratings [17], readiness to quit [22], perceived stress [23], depressive symptoms [24], social support [28], and video utilization (number of time watched DVDs and online viewing). The time-line follow-back (TLFB) [30, 31] assesses 7-day ppa, pharmacotherapy use. Participants who report 7-day ppa at the 6-month follow-up are sent a saliva collection kit to confirm abstinence via cotinine, which includes a prepaid postage envelope. Seven ng/ml distinguishes smokers from nonsmokers [32]. Those not returning saliva are considered smokers.

### Data analyses

#### Sample size and power

Previous research found 10–29% quit rates among African American quitline callers [9]. Our preliminary research showed a 7% difference in self-reported verified cessation, between participants who received only the *PTF* DVD vs. the Standard control (no additional counseling or NRT). Using the formula provided in Diggle et al. [33] (p. 31), we anticipated a 7% difference in 7-day ppa at 6-months between the *PTF* (32%) vs. control (25%) conditions, with a power of .80, alpha of .05, which requires 350 participants/condition.

#### Statistical analysis

Preliminary analyses will include descriptive statistics, analyses of variance and chi-square tests to examine group differences in baseline variables. If any differences are found, these variables will be entered as covariates in outcome analyses. Pattern mixture analyses will examine selection bias by comparing the characteristics and outcomes of participants who completed the study to those who did not both across and within study groups. Alpha will be set to .05, and adjusted for multiple comparisons if needed. Missing values are expected, and will be handled using methods such as multiple imputation, maximum likelihood estimation, and intent-to-treat analyses. The primary outcome is biochemically verified 7-day ppa (1 = cessation, 0 = smoking) at 6-months. Generalized linear mixed models (GLMMs) will test the longitudinal effect of intervention condition on abstinence, including interaction terms and adjusting for covariates. Controlling for covariates, hierarchical logistic regressions will determine the odds of abstinence at the 3-month follow-up (secondary outcome), and treatment engagement and potential moderating variables (e.g., acculturation, ethnic identify, readiness to quit) will be analyzed with analyses of covariance and hierarchical regression analyses.

### Ethics, safety, and dissemination

This study has been approved by the Case Western Reserve University Institutional Review Board (IRB). This is considered a minimal risk study, which includes state quitline services that are currently available to eligible callers, and adds a video-based intervention to two-thirds of the sample. Thus, all participants have the opportunity to receive standard care (counseling plus NRT) from the quitline. Individuals who are eligible for participation provide verbal informed consent at the time of enrollment, followed by a written information sheet that is mailed to participants. The smoking cessation intervention is delivered by the quitline per their standard operating procedures. The addition of two video-based adjuncts are not anticipated to require early stopping. Quit Coaches have been trained on the goals and procedures of the study, including screening, consenting, and incorporating the video content as appropriate into coaching calls. The principal investigators review a subset of coaching calls and provide performance feedback.

The study provides up to 2 weeks of nicotine patches, which is standard through the quitline. Using data collected from callers, Coaches provide decision support using a database-supported algorithm based on current scientific evidence and the product manufacturer use instructions for dosing. Written materials outlining the appropriate use of cessation support products also accompany the NRT shipment. Participants can contact QuitlineNC at any time if they experience any discomfort from NRT use or have questions. Participants will be directed to cease NRT use and contact their medical provider if they experience distressing or persistent side effects. Adverse events are reported to the IRB annually (for non-serious events) and within three business days (for serious events).

This trial has been approved by the Case Comprehensive Cancer Center Protocol Review and Monitoring Committee (PRMC). There is a data safety and monitoring plan, which includes multiple processes to ensure data safety. Alere Wellbeing is a HIPAA-covered entity and follows all requirements for data safety and confidentiality. In accord with our data use agreement, data transfers from the quitline to the university are completed through a secure server dedicated to the study. Restricted internal use only data are entered and stored on password-protected institutional servers and in locked files. Materials for saliva collection are labeled with participant identification numbers and non-labeled envelopes, and samples are discarded following analysis. Personally identifying information is not collected via the Internet, and para-data are used to estimate online views for the two programs. Weekly audits are conducted to confirm the entry and accuracy of data for new participants. Quarterly audits of the study, using checklists to document accuracy, are conducted independently of the investigators and sponsor. The progress of the trial is reported at 6-month intervals to the PRMC and annually to the IRB. Any personnel or protocol modifications are reported to the IRB.

The *PTF* video is available for non-commercial use through the National African American Tobacco Prevention Network. The data generated from this project is owned by Case Western Reserve University. Data collected as part of quitline registration is owned by QuitlineNC. De-identified datasets will be shared with the scientific community for research following the initial dissemination of the primary study aims, while carefully observing standards of patient privacy, confidentiality, and management of health information. Requests will be made to the PI and will also be subject to approval by QuitlineNC.

## Discussion

This semi-pragmatic RCT is the first to test the effects of a culturally specific video intervention combined with standard quitline counseling among African Americans. Innovations include implementation of the *PTF* intervention in a real-world context, examination of whether cultural specificity increases treatment engagement, consideration of individual differences that may influence treatment response, and examining the feasibility of online intervention delivery. Planned moderator analyses will also yield important insight regarding who is most benefitted by the culturally specific adjunct.

Quitlines represent a core population-based intervention with the potential to reduce barriers to help-seeking and meet the needs of underserved populations. In practice, providing culturally specific care through quitlines is a challenging task. Reasons include the extensive background and training needed, and the numerous racial/ethnic and cultural groups in the US. Moreover, tobacco use is a behavioral problem that may not seem connected to ethno-cultural factors (although it is); thus, the inclusion of a culturally specific intervention component may not be on the list of priorities for state quitlines. However, enhancing quitline services with an evidence-based approach targeting African American smokers might result in a substantial reduction in tobacco cessation disparities. One way to improve the care provided to African American quitline callers, who are disproportionately of low socioeconomic status and medically underserved, is to provide extensive training to Quit Coaches on the unique barriers often faced by this population (e.g., disproportionately high rates of health problems, major life stressors, discrimination) so that culturally specific counseling can be delivered. Another, likely more cost effective approach is to continue to provide standard care, but include the provision of an evidence-based, culturally specific enhancement. The current study offers this approach. If successful, the *PTF* intervention has the potential to be scaled at the national level.

There are several potential weaknesses of this study. The sample includes treatment-seeking tobacco smokers who may differ from non-treatment seekers. This might limit generalizability; however, the limited exclusion criteria reduce this concern. This trial is embedded within a real-world service delivery model; thus no alterations can be made in eligibility for quitline enrollment (vs. study enrollment) or the quitline counseling approach. The sample is also limited to residents in a single state, and may not represent tobacco smokers in other regions of the country. Finally, the ability to evaluate intervention effects is predicated on participants viewing the videos. As such, we are implementing an engagement protocol consisting of bi-weekly and monthly contacts to prompt intervention use and Quit Coaches are trained to refer participants to relevant areas of the videos during coaching calls.

Overall, this study will address important, unanswered questions that have the potential to change best practices for addressing tobacco use among African Americans, particularly those who use the quitline. If our hypotheses are supported, the *PTF* video could be disseminated to all 50 state quitlines, in addition to other delivery channels. The ultimate goal is to eliminate tobacco cessation disparities.

## Abbreviations

DVD: Digital video disc
GLMMs: Generalized linear mixed models
IRB: Institutional Review Board
NAQC: National American Quitline Consortium
NRT: Nicotine replacement therapy
PRMC: Protocol Review and Monitoring Committee
PTF: Pathways to Freedom: Leading the Way to a Smoke-Free Community
QuitlineNC: North Carolina State quitline
RCT: Randomized controlled trial
TLFB: Time-line follow-back
